# Exploring the Impact of Different Processing Techniques on Quality and Flavor Characteristics in Hoki Steak Soups

**DOI:** 10.1002/fsn3.4616

**Published:** 2024-11-28

**Authors:** Xiaoqing Miao, Jing Li, Shuang Li, Guodong Li, Xiuping Dong, Pengfei Jiang

**Affiliations:** ^1^ SKL of Marine Food Processing & Safety Control, National Engineering Research Center of Seafood, School of Food Science and Technology Dalian Polytechnic University Dalian China; ^2^ Qingdao Yihexing Foods Co. Ltd Qingdao Shandong China

**Keywords:** e‐nose, gc‐ims, hoki steak soup, processing methods, volatile compounds

## Abstract

This study investigated the effect of different processing methods (boiling, oil boiling, and stir frying) on the flavor of hoki steak soups. The quality of different fillet broths was explored by pH, Thiobarbituric acid reactive substances (TBARS), and color. E‐nose, E‐tongue, and gas chromatography‐ion mobility spectrometry (GC‐IMS) combined with free amino acids (FAAs) were used to analyze the flavor of hoki steak soups. Key compounds were screened by relative odor activity value (ROAV). The variable influence on projection (VIP) was used to identify the differential compounds. The E‐nose and E‐tongue were able to distinguish hoki steak soups. A total of 47 volatile compounds were characterized by GC‐IMS. n‐Pentanal (M) was the key component for the difference between hoki steak soups. There were six substances with ROAV ≥ 1. The results showed that the hoki steak soup boiled for 2 h in the stir‐frying group had a lower fishy odor.

## Graphical abstract

This study investigated the effect of different processing methods (boiling, oil boiling, stir‐frying) on the flavor of hoki steak soups, and the results showed that the hoki steak soup boiled for 2 hours in the stir‐frying group had a better flavour andlower fishy odor.

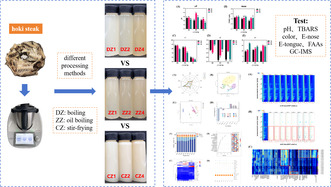

## 1 Introduction

Cod is abundant in high‐quality protein, ranking among the most plentiful fish species globally (Ma et al. 2019). New Zealand hoki ( *Macruronus novaezelandiae* ) is particularly abundant in New Zealand waters and is a prominent commercial species (Koot et al. 2021). New Zealand hoki, known for its tender, white flesh, and minimal spines, is regarded as a high‐quality, safe marine fish (Bah et al. 2016). It is exceptionally nutritious, providing a rich source of proteins, vitamins, minerals, and omega‐3 fatty acids (Elsebaie et al. 2022). New Zealand hoki is usually processed into fish pieces and fillets, and one of its main by‐products is fish steak. Fish steaks are actually fish bones, with a large amount of fish flesh remaining between the bones, accounting for about 15% of the total weight of the fish (Calderon‐Garcia et al. 2012). The rapid expansion of the aquatic processing industry has led to the annual generation of millions of tons of fish by‐products worldwide (Siewe, Kudre, and Narayan 2021). These by‐products are abundant in nutrients and functional components, including bioactive peptides, collagen, gelatin, and others (Hong et al. 2019; Wang et al. 2022; Mirzapour‐Kouhdasht et al. 2021). Fish steak, primarily processed into bone meal for feed or fertilizer, rarely undergo further processing, which not only causes waste of resources but also imposes a certain burden on the environment. Therefore, maximizing the high‐value utilization of fish processing by‐products has emerged as a pivotal challenge for the fish processing industry (Ozuna et al. 2015).

Flavor comprises a complex array of odors and taste molecules emanating from volatile and nonvolatile compounds (Zhang et al. 2024), representing one of the foremost indicators of food quality (Zeng et al. 2020). Currently, chromatography‐mass spectrometry and electronic nose (E‐nose) are the most important methods for the detection of volatile compounds (Jin et al. 2023). Gas chromatography‐ion mobility spectrometry (GC‐IMS) stands out as a swift technique for volatile component determination (Li et al. 2024), which fully combines the high‐resolution capability of GC technology with the high discrimination capability of IMS technology (Chen et al. 2024), and it has a good prospect for application in the field of food analysis (Du et al. 2021) and has been widely used in the detection and quantitative analysis of volatile compounds in aquatic products and meat (Zhang et al. 2020). To improve the sensitivity of odor identification, electronic nose technology was introduced. E‐nose collects and identifies single or complex gases to predict odor attributes through an array of odor sensors (Qiu and Wang 2017; Zou et al. 2024) and has the benefits of being comprehensive, fast, environmentally friendly, and simple sample pretreatment (Lu et al. 2022), which can effectively identify and detect volatile compounds in food products.

Fish soup is a complex reaction system, and different processing methods may affect its quality and flavor. Most of the current studies on the processing methods of fish soup mainly focus on the preparation of fish soup by frying in oil and then boiling, and few studies have investigated the effects of processing methods such as boiling in water directly on the quality and flavor of fish soup. In addition, the quality of fish soup further affects the flavor of fish soup, and the two are interrelated. However, the current research tends to be unilateral. Meanwhile, the existing studies usually select whole fish or fish head as the raw material for making fish soup, and there is no study on the preparation of fish soup using fish steak as the raw material. Therefore, this study took fish steaks as the research object to comprehensively investigate the effects of different processing methods on the quality and flavor of fish steak soup. The effects of different processing methods on the quality of hoki steak soups were investigated by measuring pH, TBARS, and color. The differences of volatile compounds in the hoki steak soups were further analyzed by using electronic nose (E‐nose) and GC‐IMS, and the fingerprints of the hoki steak soup prepared by three different processing methods were constructed. In addition, the effects of different processing methods on the taste of hoki steak soups were investigated based on free amino acids (FAAs) and electronic tongue (E‐tongue) results. This study aims to provide a theoretical reference for the optimization of the processing technology of fish soup as well as to provide guidance for the comprehensive utilization of fish by‐products, which is of great significance to improve the high value utilization of fish by‐products.

## 2 Material and Methods

### 2.1 Materials and Chemicals

Hoki steak was obtained from Qingdao Yihexing Foods Co. Ltd. (Qingdao, Shandong, China), and each steak was approximately 50 cm in length and weighed approximately 350 g. The steaks were transported to the laboratory and frozen at −40°C for subsequent use.

Wahaha mineral water and Jinlongyu soy bean oil were purchased from a local supermarket. 2‐Thiobarbituric acid and trichloroacetic acid (analytically pure, AR) were acquired from Shanghai McLean Biochemical Technology Co. Ltd. (Shanghai, China). 3‐Hydrochloric acid (analytically pure, AR) was purchased from Sichuan Xilong Science Co. Ltd. (Sichuan, China). Salicylic acid was purchased from Tianjin Damao Chemical Reagent Co. Ltd. (Tianjin, China).

### 2.2 Preparation of Hoki Steak Soups

First, the hoki steaks were defrosted under running water, then the steaks were cleaned to remove impurities and blood and subsequently chopped into pieces (30 ± 5 g) and put into an electric food processor (multifunctional food processor) (TM6‐1, Thermomix, Germany) to make hoki steak soups. The specific processing methods were shown below:

1. Boiled group hoki steak soup (DZ): Hoki steak soup in the boiled group (DZ) was prepared by combining 500 g of hoki steak with 1600 g of water in a 5:16 ratio (w/w). The mixture was heated to 100°C with a rotation speed of 0.5 r/s until boiling, followed by a reduction of temperature to 95°C with the same rotation speed for simmering over low heat for 1, 2, and 4 h, denoted as DZ 1, DZ 2, and DZ 3, respectively.
2. Boil hoki steak soup with oil (ZZ): Following a ratio of hoki steak, oil, and water at 5:1:15 (w/w/w), 500 g of hoki steak, 100 g of vegetable oil, and 1500 g of water were combined. The mixture was heated to 100°C with a rotational speed of 0.5 r/s until boiling, then simmered at 95°C with the same rotational speed for 1, 2, and 4 h, labeled as ZZ 1, ZZ 2, and ZZ 3, respectively.
3. Stir‐fried hoki steak soup (CZ): Using the same ratio of hoki steak, oil, and water (5:1:15, w/w/w), 500 g of hoki steak and 100 g of vegetable oil were stir‐fried at 120°C with a rotational speed of 1.0 r/s for 0.5 h. Following this, 1500 mL of water were added, and the mixture was cooked at 100°C with a rotational speed of 0.5 r/s until boiling. Finally, it was simmered at 95°C with the same rotational speed for 0.5, 1.5, and 3.5 h, labeled as CZ 1, CZ 2, and CZ 3, respectively.

Once hoki steak soup has cooled to room temperature, strain twice and set aside in a container. For the preparation of hoki steak soups, three different batches of hoki steaks were selected, and then samples from each batch of hoki steak soup were selected for subsequent experiments.

### 2.3 pH

Hoki steak soup (30 mL) was transferred into a 50‐mL beaker, followed by pH determination using a pH meter (PB‐10, Sartorius, Germany). pH readings were recorded when the display stabilized. Three parallel measurements were conducted for each sample, and their average was calculated.

### 2.4 Thiobarbituric Acid Reactive Substances (TBARS)

The TBARS were determined by the method of Wang et al. (2024) with some modifications. Each sample (0.5 mL) was placed into a 15‐mL centrifuge tube, followed by the addition of 2.5 mL of TBARS test solution (15% TCA and 0.375% TBARS dissolved in 0.25 mol/L HCl). After a 15‐min boiling bath, the sample was cooled to room temperature and then centrifuged at 5000 r/min at 4°C for 10 min using a high‐speed refrigerated centrifuge (CR22N, Hitachi, Japan) equipped with an R15A rotor. Subsequently, the absorbance of the supernatant at 532 nm was then measured, and the value was multiplied by 2.77 to give the TBARS result.

### 2.5 Color Measurement

The lightness (L*), redness (a*), and yellowness (b*) values of hoki steak soups were measured using a Color Meter (Ultra Scan PRO, Hunter Lab, USA) in TTRN total transmission mode. Prior to measurement, the color meter was calibrated using a standard blackboard and white plate. Subsequently, the hoki steak soup samples were analyzed, and their L*, a*, and b* values were recorded.

### 2.6 E‐Nose Determination

Volatile compounds in hoki steak soups prepared by three processing methods were analyzed using an electronic nose (PEN3.0, Airsense, Germany). Five milliliters of hoki steak soup sample were placed into a 20‐mL headspace bottle and left at room temperature for 30 min before analysis. The detection parameters were configured as follows: sample preparation time, 1 s; presampling time, 5 s; detection time, 100 s; auto‐zero time, 10 s; sensor cleaning time, 60 s; carrier gas flow rate, 300 mL/min; injection volume, 300 mL/min. Data analysis was performed from 83 to 87 s using the Winmuster software provided with the electronic nose (Wei et al. 2024).

### 2.7 E‐Tongue Determination

Hoki steak soup flavors were analyzed using an E‐tongue (TS‐5000Z; Insent, Japan). Measurement method following Wang et al. (2024) with some modifications. 10 mL of sample were diluted with 100 mL of deionized water, centrifuged for 10 min (8000 rpm, 4°C), and the supernatant was collected and filtered to obtain it. Then, 40 mL of the filtrate was pipetted into the E‐tongue's special chamber, and then the samples were measured using the machine.

### 2.8 Determination of Free Amino Acid (FAA)

The method of Li et al. (2015) was modified to determine the content of the free amino acid. A 1 mL sample was placed in a 15‐mL centrifuge tube, 1 mL of 5% salicylic acid was added, sonicated for 10 min, and centrifuged at 5000 rpm for 10 min, and the supernatant was collected. The supernatant was filtered through a 0.45 μm filter and analyzed by an amino acid analyzer (Biochrom 30+; Biochrom, England).

### 2.9 GC‐IMS Analysis

Volatile compounds in hoki steak soup were detected using GC‐IMS (Flavourspec, G.A.S., Dortmund, Germany) according to the method previously reported by Miao et al. (2023). The headspace conditions were as follows: 1 mL of sample was equilibrated in a headspace vial (80°C, 15 min). The injection needle temperature was 85°C and the injection volume was 500.0 μL. The IMS conditions were as follows: A column (MXT‐5, 15 m, 0.53 mm, 1.0 μm, RESTEK, USA) with a column temperature of 60°C. The flow rate varies as follows: Initially, 2.0 mL/min was maintained for 2 min and then increased linearly to 10 mL/min over 10 min, followed by a linear increase to 100 mL/min over 20 min, and then a linear increase to 150 mL/min over 30 min.

### 2.10 Characteristic Flavors Analysis

The ROAV approach was employed for assessing the flavor contribution of volatile compounds. In this method, ROAV values ≥ 1 indicate characteristic flavor components, while those ranging from 0.1 to < 1 denote important flavor components. The component with the highest overall flavor contribution was designated as ROAVstan = 100. Subsequently, the ROAV of other volatile compounds in the sample was determined using the following equation:

(1) $$\text{ROAV}=\frac{C_{i}}{C_{\text{stan}}}\times \frac{T_{\text{stan}}}{T_{i}}\times 100$$

where *C*_i_ represents the relative content of volatile compounds (%); *T*_i_ is the sensory threshold of compounds in water (μg/kg); and *C*_stan_ and *T*_stan_ represent the relative content and sensory threshold of compounds with the highest contribution, respectively.

### 2.11 Statistical Analysis

Experiments were conducted in triplicate, and results were presented as mean ± standard deviation. Statistical analysis was performed through analysis of variance (ANOVA) using SPSS 26.0 software (IBM, Armonk, New York, USA), with significance set at *p* < 0.05. Data handling, statistical analysis, and visualization were executed using Excel 2010, GraphPad, and MetaboAnalyst for heatmaps and VIP plots, respectively.

## 3 Results and Discussion

### 3.1 Analysis of pH

**FIGURE 1:**
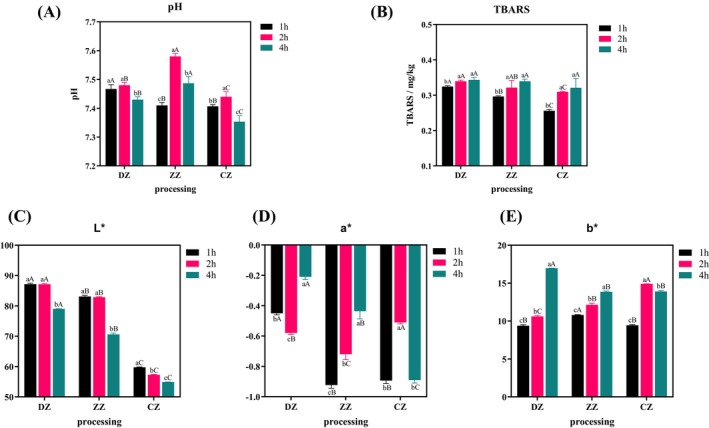
Effect of different processing methods on pH (A), TBARS (B), L* (C), b* (D), and a* (E) of hoki steak soups. DZ: Boiled group hoki steak soup, ZZ: Boil hoki steak soup with oil, CZ: Stir‐fried hoki steak soup. 1, 2, and 3 indicated 1, 2, and 4 h of simmering.

pH is a crucial indicator for evaluating fish soup deterioration and spoilage. The pH changes in hoki steak soups prepared by different processing methods were shown in Figure 1A. The pH values of all three groups of hoki steak soups showed a tendency of increasing and then decreasing with the extension of the simmering time, which may be due to the gradual reduction of numerous free acidic groups in the proteins due to the denaturation by heating, which led to a rapid increase in their pH (Huang et al. 2011). With the extension of soup cooking time, the content of oil in hoki steak soup increased, which reduced the oxygen content in hoki steak soup, thus promoting the decomposition of glycogen in hoki steak soup into lactic acid, and the lactic acid content increased compared with before, so the pH decreased. At the 1st h, the difference in pH between ZZ and CZ was not significant (*p* > 0.05), while both were significantly lower (*p* < 0.05) compared to the pH of DZ. At the 2nd and 4th hours, there was a significant difference (*p* < 0.05) in the pH of hoki steak soup prepared by the different processing methods, with the pH of ZZ being significantly higher (*p* < 0.05) than that of DZ, while the pH of CZ was significantly lower (*p* < 0.05) than that of DZ in both cases.

### 3.2 Analysis of TBARS

The TBARS value is the result of the reaction between malondialdehyde (MDA) formed by secondary oxidation of lipids and thiobarbituric acid under certain conditions (De Mey et al. 2017). The TBARS values of hoki steak soups prepared by different processing methods were shown in Figure 1B. The TBARS values of all three groups of hoki steak soup showed a significant increase (*p <* 0.05) with the extension of the simmering time, and the larger the value indicated the more serious degree of fat oxidation (Tinagli et al. 2023), which showed that lipid oxidation was continuously occurring in hoki steak soup. At identical simmering durations, significant differences (*p* < 0.05) existed in TBARS values among the three hoki steak soup groups. Specifically, CZ 1, CZ 2, and CZ 3 exhibited lower values compared to the other two groups, indicating a lesser degree of overall fat oxidation in CZ. This discrepancy may stem from reduced malondialdehyde content resulting from its dissolution in the stir‐frying process or its formation of adducts with proteins.

### 3.3 Analysis of Color

Color is an intuitive factor in evaluating soup quality. The results indicated a gradual decrease in the L* value of hoki steak soups across all three groups with increasing boiling time, indicating a decrease in transmittance (Figure 1C). Meanwhile, the b* value significantly increased over time (*p < 0.05*) (Figure 1D). This trend was primarily attributed to the dissolution of proteins and fats in the fillets during prolonged boiling, resulting in decreased soup brightness. Additionally, emulsification of fat particles contributed to the heightened yellowness of hoki steak soup. In DZ, the a* values initially decreased and then increased as simmering time prolonged, whereas in ZZ and CZ, there was a continuous increase in a* values over time. Notably, all three groups of hoki steak soups exhibited negative a* values, indicating low redness (Figure 1E). At identical simmering times, the L* values significantly varied among the three groups of hoki steak soups (*p <* 0.05), with DZ exhibiting the highest L* value and CZ the lowest. This discrepancy primarily stemmed from the substantial rise in oil content in hoki steak soup postfrying. Furthermore, prolonged simmering led to the emulsification of proteins from fish bones with oils and fats, resulting in a milky white broth.

### 3.4 Analysis of E‐Nose

**FIGURE 2:**
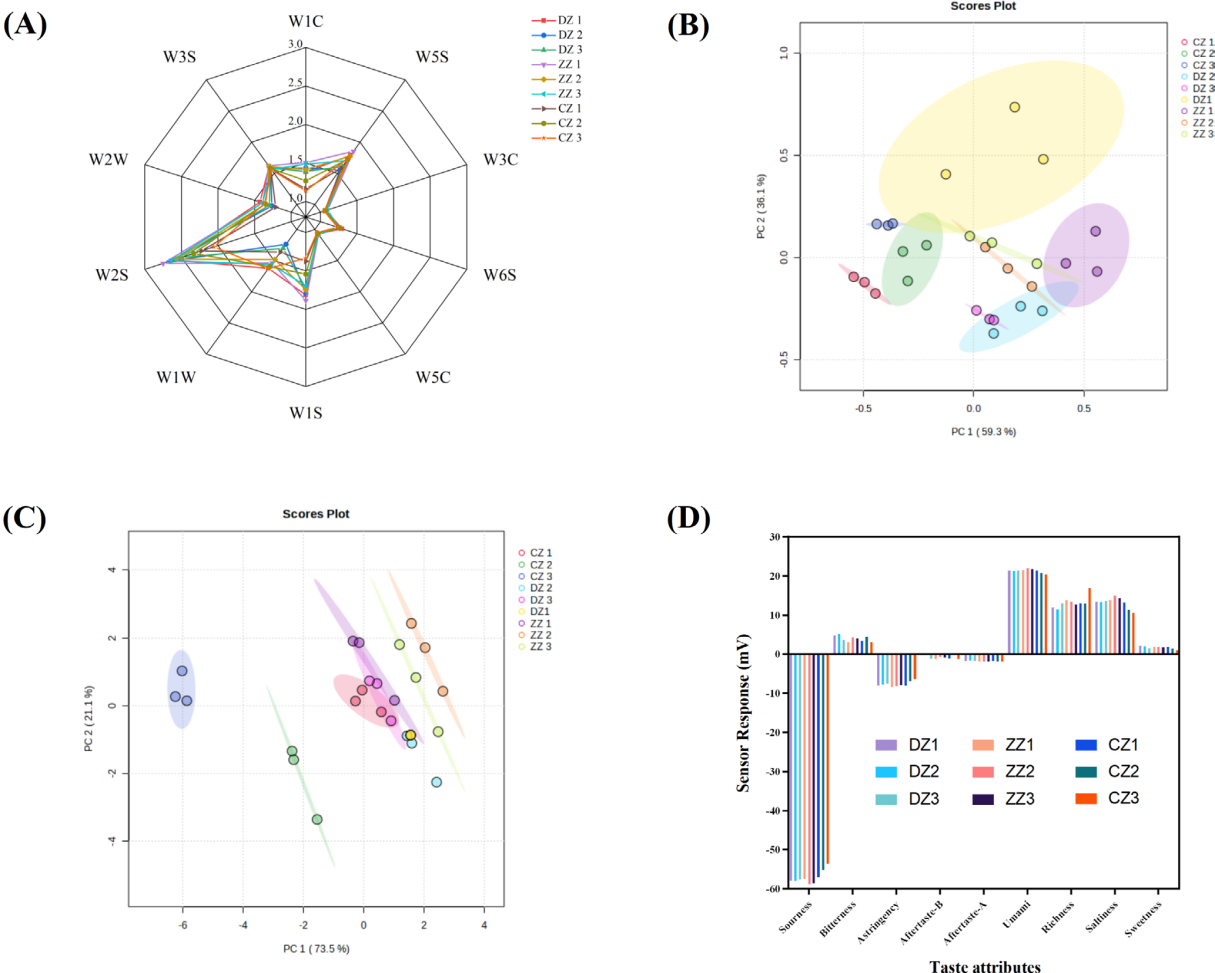
Radar map (A) and PCA (B) and of E‐nose; PCA (C), histogram of taste indicators (D) of E‐tongue. DZ, ZZ, and CZ are the same as Figure 1.

E‐nose was employed to assess the flavor of hoki steak soup prepared by different processing methods. The radargram of the E‐nose is shown in Figure 2A. As shown, the response values of W2S (alcohols, aldehydes, and ketones), W5S (oxides of nitrogen), and W1S (methyl) were significantly higher than those of other sensors, and among them, the W2S sensor has the highest response value for hoki steak soups, which indicated that the content of alcohols, aldehydes, ketones, and oxides of nitrogen, as well as methyl in hoki steak soups were high.

PC1 and PC2 contributed 59.3% and 36.1%, respectively, to a cumulative contribution of 95.4%, indicating significant variation in the odor profiles of hoki steak soups prepared by different processing methods. The CZ appeared predominantly on the left side of the plot, distinct from the DZ and ZZ, suggesting significant differences in odor profiles. Conversely, DZ and ZZ exhibited notable overlap, implying similarities in odor characteristics between them. These findings suggested that pan‐frying followed by simmering may enhance the release of odor compounds in hoki steak soups. In addition, there was no overlap and intersection of hoki steak soup samples with different simmering times among the three processing methods in the graph, suggesting that the differences in the odor of hoki steak soups with different simmering times were significant and could be well differentiated.

### 3.5 Analysis of E‐Tongue

PCA analysis was performed on the data from the E‐tongue (Figure 2C). PC1 and PC2 collectively accounted for 94.6% of the variance, demonstrating the efficacy of the E‐tongue combined with PCA in discriminating between hoki steak soups prepared by different processing methods. The PCA plot revealed distinct separation between CZ 2, CZ 3, and the remaining samples, suggesting a pronounced divergence in taste profiles of these hoki steak soups. In addition, there was no overlap between ZZ 2 and ZZ 3 and the other samples, which were similar to the three sets of samples from the DZ and more distant from CZ 2 and CZ 3. It is worth noting that both ZZ 1 and CZ 1 had more obvious overlapping parts with the three samples from the DZ group, suggesting that there was not much difference in the taste of the hoki steak soups prepared by different processing techniques within 1 h of simmering. The histogram (Figure 2D) of the taste values of the hoki steak soup samples versus the reference solution showed that the sour, astringent, and bitter aftertaste in the hoki steak soups were all at a point below the no‐taste point, indicating that these flavors were not present in the hoki steak soups. Differences in flavor among several hoki steak soups were mainly in taste indicators such as salty, richness, bitterness, and freshness. In terms of freshness and saltiness, the ZZ group was the largest, and the richness of CZ 3 was the largest and much larger than that of the other samples.

### 3.6 Analysis of FAAs

Differences in the types and contents of FAAs conferred different flavor characteristics to the hoki steak soups. The types and contents of FAAs in the hoki steak soups prepared by different processing methods differed significantly (Table 1). Except for ZZ 2 and CZ 3, 17 FAAs were detected in all the other hoki steak soups, which mainly consisted of three major categories, namely fresh amino acids, sweet amino acids, and bitter amino acids. The largest percentage of bitter amino acids, followed by sweet amino acids, and the least amount of fresh amino acids were found in all the hoki steak soups. However, the contents of all bitter amino acids were much less than their taste thresholds. It has been shown that the content of bitter amino acids is related to bitterness and that bitter amino acids below their threshold have the effect of enhancing freshness and increasing the taste (Zhao, Schieber, and Ganzle 2016).

**TABLE 1:** FAAs content of hoki steak soups prepared by different processing methods.

FAAs	Thresholds (mg/100 mL)	Free amino acid content (μg/mL)								
		DZ 1	DZ 2	DZ 3	ZZ 1	ZZ 2	ZZ 3	CZ 1	CZ 2	CZ 3
Asp	100	1.55 ± 0.04^b,C^	3.44 ± 0.28^a,A^	0.94 ± 0.00^c,A^	1.74 ± 0.00^b,B^	4.11 ± 0.16^a,A^	ND^c,B^	1.9 ± 0.03^b,A^	3.63 ± 0.03^a,A^	ND^c,B^
Glu	30	15.71 ± 0.02^b,A^	18.38 ± 0.05^a,C^	3.17 ± 0.05^c,B^	11.36 ± 0.04^b,C^	23.67 ± 0.43^a,A^	1.70 ± 0.04^c,C^	11.64 ± 0.07^b,B^	19.98 ± 0.19^a,B^	8.24 ± 0.05^c,A^
Thr	260	7.23 ± 0.01^b,C^	9.42 ± 0.06^a,C^	9.39 ± 0.02^a,A^	9.55 ± 0.08^b,B^	11.30 ± 0.01^a,A^	5.53 ± 0.03^c,C^	10.43 ± 0.20^a,A^	9.90 ± 0.18^b,B^	8.48 ± 0.03^c,B^
Ser	150	7.79 ± 0.03^c,C^	10.62 ± 0.12^a,B^	9.20 ± 0.04^b,A^	9.02 ± 0.33^b,B^	12.11 ± 0.23^a,A^	2.83 ± 0.02^c,B^	10.52 ± 0.29^a,A^	7.37 ± 0.01^b,C^	1.51 ± 0.37^c,C^
Gly	130	9.50 ± 0.10^b,C^	12.00 ± 0.41^a,A^	11.38 ± 0.09^a,B^	14.19 ± 0.18^a,A^	11.18 ± 0.23^b,A^	9.02 ± 0.15^c,C^	11.67 ± 0.02^b,B^	11.88 ± 0.00^a,b,A^	12.3 ± 0.24^a,A^
Ala	60	20.65 ± 0.12^b,B^	24.66 ± 0.29^a,B^	24.59 ± 0.08^a,B^	22.42 ± 0.37^b,A^	23.71 ± 0.10^a,B^	16.11 ± 0.10^c,C^	23.05 ± 0.33^b,A^	25.86 ± 0.50^a,A^	25.98 ± 0.59^c,A^
Pro	300	5.46 ± 0.02^a,A^	5.45 ± 0.01^a,B^	4.64 ± 0.01^b,A^	5.01 ± 0.00^b,B^	5.55 ± 0.00^a,A^	ND^c,B^	5.05 ± 0.06^b,B^	5.47 ± 0.02^a,B^	ND^c,B^
Cys	—	28.84 ± 0.46^b,A^	32.74 ± 1.12^a,A^	29.18 ± 0.28^b,B^	29.16 ± 0.38^a,A^	27.70 ± 0.30^a,B^	28.17 ± 0.60^a,B^	29.49 ± 0.50^b,A^	28.66 ± 0.22^c,B^	30.72 ± 0.06^a,A^
Val	40	12.64 ± 0.36^a,B^	12.65 ± 0.84^a,A^	12.33 ± 0.37^a,B^	12.55 ± 0.47^b,B^	13.78 ± 0.05^a,A^	8.05 ± 0.13^c,C^	14.45 ± 0.12^a,A^	12.55 ± 0.16^a,A^	13.79 ± 0.33^b,A^
Met	30	7.09 ± 0.16^a,C^	7.50 ± 0.33^a,A^	7.63 ± 0.02^a,B^	7.45 ± 0.09^b,B^	7.82 ± 0.10^a,A^	5.78 ± 0.06^c,C^	7.93 ± 0.02^b,A^	7.16 ± 0.00^c,A^	12.43 ± 0.05^a,A^
Ile	90	3.95 ± 0.12^a,B^	3.30 ± 0.11^b,C^	3.36 ± 0.09^b,B^	4.08 ± 0.39^b,B^	5.34 ± 0.26^a,A^	1.99 ± 0.08^c,C^	5.10 ± 0.05^b,A^	4.03 ± 0.25^c,B^	5.77 ± 0.16^a,A^
Leu	190	8.70 ± 0.09^b,C^	8.81 ± 0.03^b,C^	8.48 ± 0.14^b,B^	9.33 ± 0.03^b,B^	11.33 ± 0.13^a,A^	4.67 ± 0.02^c,C^	10.61 ± 0.14^a,A^	9.84 ± 0.05^b,B^	11.04 ± 0.30^a,A^
Tyr	260	6.63 ± 0.10^a,A^	6.17 ± 0.02^b,A^	4.80 ± 0.15^c,A^	5.29 ± 0.03^a,C^	4.64 ± 0.04^a,B^	2.91 ± 0.37^b,B^	5.57 ± 0.02^b,B^	4.11 ± 0.04^c,C^	5.20 ± 0.01^a,C^
Phe	90	7.40 ± 0.43^a,A^	3.73 ± 0.16^b,B^	3.03 ± 0.14^b,B^	2.82 ± 0.11^b,C^	2.78 ± 0.05^b,C^	3.37 ± 0.16^a,B^	5.81 ± 0.41^b,B^	5.80 ± 0.07^b,A^	23.27 ± 0.47^a,A^
His	20	2.99 ± 0.08^a,C^	2.26 ± 0.27^b,B^	3.02 ± 0.08^a,B^	4.76 ± 0.16^a,A^	3.21 ± 0.07^b,A^	0.98 ± 0.11^c,C^	4.30 ± 0.05^a,B^	3.66 ± 0.05^a,b,A^	3.92 ± 0.10^b,A^
Lys	50	17.58 ± 0.40^c,B^	19.57 ± 0.06^b,B^	20.55 ± 0.00^a,B^	19.82 ± 0.55^b,A^	21.04 ± 0.19^a,A^	14.48 ± 0.03^c,C^	19.13 ± 0.20^a,A^	18.97 ± 0.00^a,B^	25.12 ± 0.04^a,A^
Arg	50	4.54 ± 0.17^a,A^	4.37 ± 0.64^a,B^	4.20 ± 0.06^a,A^	5.10 ± 0.13^b,A^	6.82 ± 0.14^a,A^	2.73 ± 0.89^c,A^	4.23 ± 0.62^a,A^	4.43 ± 0.07^a,C^	ND^c,B^
TFAAs		168.27 ± 0.04^b,C^	185.06 ± 0.04^a,B^	159.88 ± 0.01^c,B^	173.66 ± 0.02^c,B^	196.09 ± 0.06^b,A^	108.32 ± 0.03^a,C^	180.89 ± 0.07^c,A^	183.29 ± 0.02^b,C^	187.76 ± 0.11^a,A^

*Note:* Lowercase represented the variability between samples with different processing times for the same processing methods and uppercase represented the variability between samples with different processing methods for the same processing time.

Abbreviations: ND, not detected; TFAAs, total FAAs.

In the 1 and 4 h hoki steak soups, the total free amino acid content of the CZ group was higher than that of the DZ and ZZ groups, which was attributed to the fact that the stir‐frying process would change the texture of the fish with a large degree of fragmentation, which was more conducive to the dissolution of free amino acids. However, in the 2 h hoki steak soups, the total free amino acid content of the CZ group was much smaller than that of the ZZ and DZ groups, probably because under the same boiling time, the frying process reacted with the fat to a greater extent, generating rich flavor substances and reducing the free amino acid content.

The total amino acid content of ZZ 2 was the highest, mainly because of the high content of fresh and sweet amino acids, aspartic acid (Asp) and glutamic acid (Glu), which are MSG‐like freshness substances (Chen et al. 2022), which were the highest in ZZ 2. The E‐tongue results also proved that the freshness of ZZ 2 was the greatest. In addition, we found that the content of fresh amino acids in the hoki steak soups of the three groups decreased significantly with the prolongation of the simmering time, especially aspartic acid (Asp), which was not detected in ZZ 3 and CZ 3, indicating that the hoki steak soups boiled for a long period of time had less fresh flavor.

### 3.7 Analysis of GC‐IMS

#### 3.7.1 GC‐IMS 2D Spectral Analysis of Hoki Steak Soups

**FIGURE 3:**
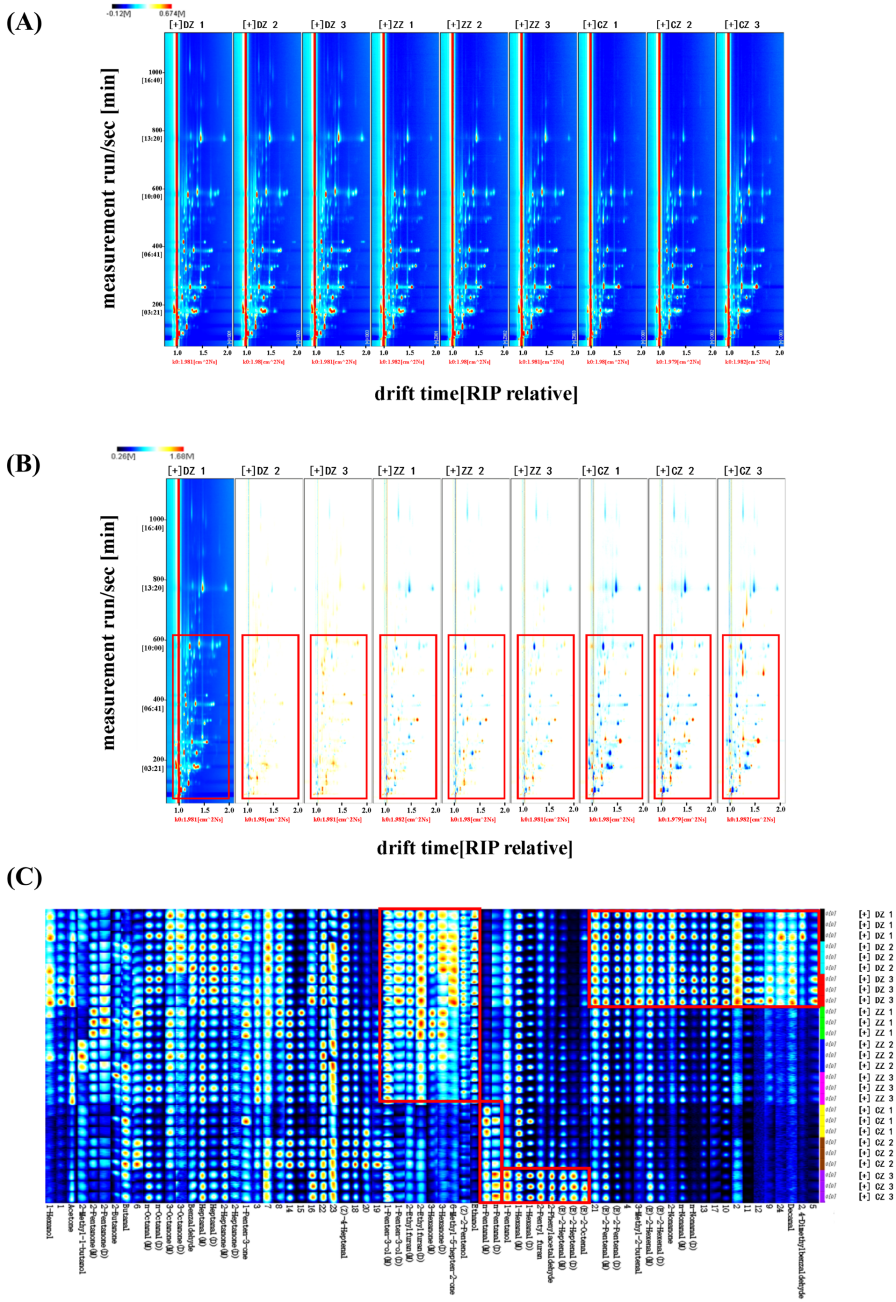
GC‐IMS analysis of hoki steak soups. 2D‐topographic plots (A) the difference comparison topographic plots (B) and fingerprints of volatile compounds (C).

The GC‐IMS two‐dimensional spectra of volatile compounds in hoki steak soups were shown in Figure 3A. There were significant differences in the volatile compounds in hoki steak soups prepared by different processing methods. To better reflect the differences in the content of volatile compounds between different hoki steak soup samples, the difference comparison spectra between samples were plotted using DZ 1 as a reference, as shown in Figure 3B. The shade of red denotes a substance with a higher concentration than the reference sample, while the blue color denotes a material with a lower concentration than the reference sample (Zhao et al. 2022). It could be seen that there was a tendency for the relative content of some volatile compounds to increase or decrease in different processes, and in particular the volatile compounds in CZ showed a more pronounced difference from those in DZ and ZZ (shown in the red box in Figure 3B). The probable reason for this was that the frying process alters the flavor of hoki steak soups by destroying the tissue structure of the fish, which resulted in the further release of flavor precursors such as proteins and fats (Wu, An, and Xiong 2023).

#### 3.7.2 Fingerprint Profiling of Hoki Steak Soups

The fingerprint spectrum of volatile compounds of hoki steak soups prepared by different processing methods is shown in Figure 3C. In the plot, each row represents all signal peaks selected from a sample of hoki steak soup, and each column represents signal peaks from the same volatile organics in different soup samples, and the darker the red color of the signal, the greater the relative mass concentration. The graph shows the full VOC information for each sample as well as the variations in VOCs between samples. 2,4‐Dimethylbenzaldehyde, Decanal, n‐Nonanal, 2‐Nonanone, (E)‐2‐Hexenal, 3‐Methyl‐2‐butenal and (E)‐2‐Pentenal were significantly higher in DZ than in ZZ and CZ; n‐Pentanal was significantly more abundant in CZ than in DZ and ZZ; ethanol, (Z)‐2‐Pentenol, 6‐Methyl‐5‐hepten‐2‐one, 3‐Hexanone, 2‐Ethylfuran, and 1‐Penten‐3‐ol were significantly less abundant than in DZ and ZZ; Trans‐2‐octenal, trans‐2‐heptenal, 2‐phenylacetaldehyde, 2‐pentylfuran, hexanal, and pentanol (E)‐2‐Octenal, (E)‐2‐Heptenal, 2‐Phenylacetaldehyde, 2‐Pentyl furan, 1‐Hexanal, and 1‐Pentanol were the most abundant substances in CZ 3. These key volatile flavor compounds may have some influence on the odor substances of hoki steak soups prepared by different processing methods. Overall, there was some variability in the volatile compositions of hoki steak soups prepared by the three processing methods.

#### 3.7.3 Qualitative and Quantitative Analysis of Volatile Compounds in Hoki Steak Soups

**FIGURE 4:**
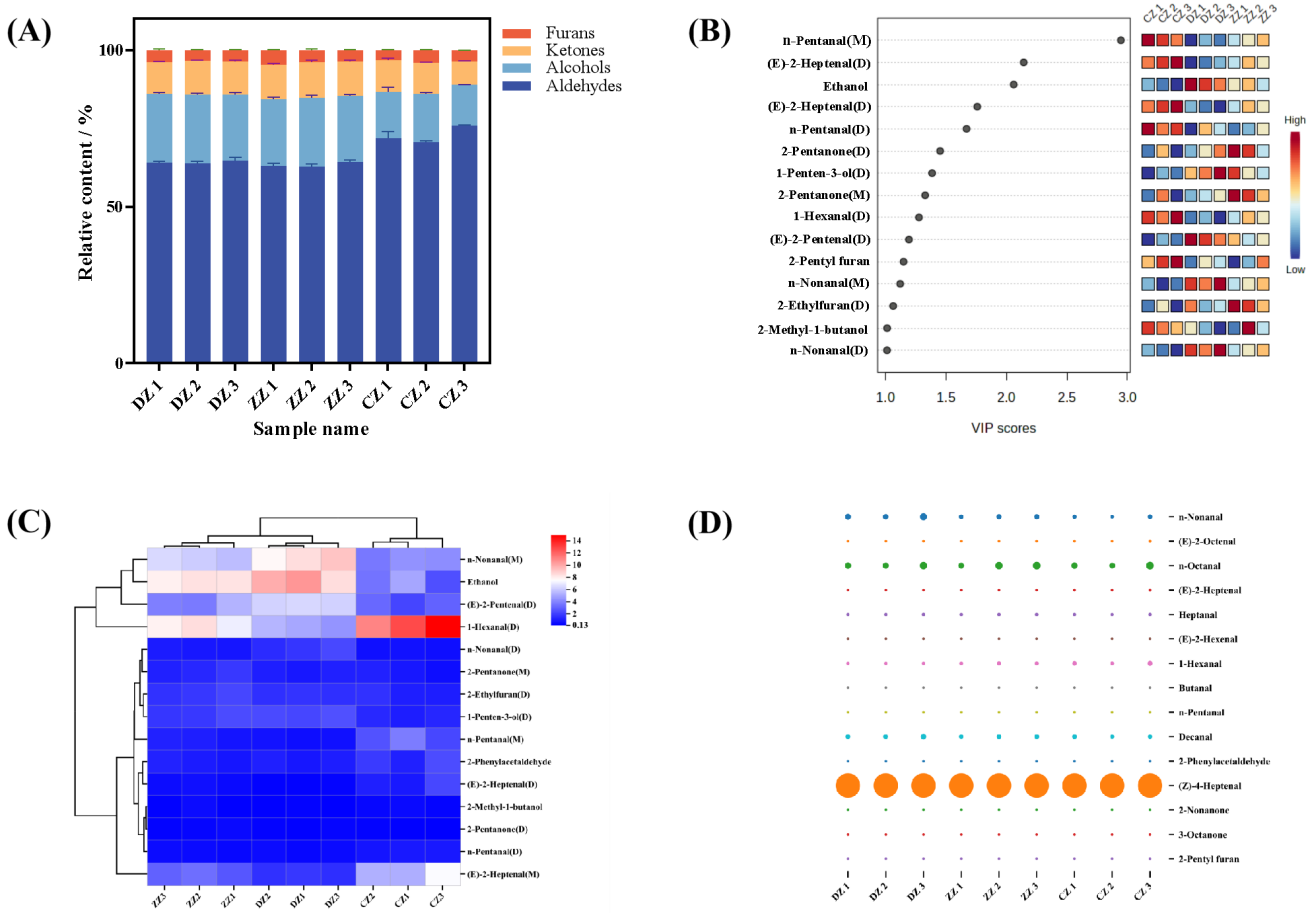
Plot of percentage of volatile compounds (%) (A), VIP > 1 (B), heatmap of volatile compounds with VIP > 1 (C) and (D) bubble plots for ROAV ≥ 0.1. DZ, ZZ and CZ are the same as Figure 1.

To further analyze and compare the changes of volatile compounds in hoki steak soups prepared by different processing methods, two‐dimensional qualitative analysis was carried out by using the built‐in NIST and IMS databases of the software, and the results were shown in Table 1. Forty‐seven volatile compounds (monomers and dimers for some of the substances) were unambiguously characterized by using GC‐IMS, which mainly included 24 aldehydes, 7 alcohols, 13 ketones, and 3 furans. Treating the total content of all volatile compounds in each sample as 100%, plot the percentage stacking of volatile compounds as shown in Figure 4A. Aldehydes predominated in hoki steak soups, constituting 60%–75% of the total volatile content and significantly surpassing other volatile flavor substances (*p* < 0.05). Most aldehydes had lower thresholds, significantly influencing the soup's flavor (Zeng et al. 2020). The aldehyde content in CZ significantly exceeded that in DZ and ZZ, implying that frying facilitates enhanced aldehyde volatilization. Alcohols typically derive from the oxidation of unsaturated fatty acids (Li et al. 2021), with CZ exhibiting significantly lower levels compared to DZ and ZZ (*p* < 0.05). This could be attributed to accelerated fat oxidation and the Maillard reaction during frying, inhibiting enzymatic oxidation and carbonyl‐ammonia reduction, consequently reducing the relative alcohol content (Molina‐Garcia et al. 2017).

#### 3.7.4 Determination of Characteristic Volatile Compounds

Further analysis was conducted to identify crucial volatile compounds in hoki steak soups using ROAV. Substances with 0.1 ≤ ROAV < 1 are usually considered to play a role in modifying the overall flavor, and substances with ROAV ≥ 1 are the key components in the samples (Liu et al. 2023). Fifteen volatile compounds with ROAV values ≥ 0.1 (Table 2S) were identified from three hoki steak soup groups, with their corresponding bubble diagrams depicted in Figure 4D. Six compounds with ROAV values ≥ 1, all aldehydes (n‐Nonanal, n‐Octanal, Heptanal, 1‐Hexanal, Decanal, and (Z)‐4‐Heptenal), were identified. Among these, (Z)‐4‐Heptenal was the predominant contributor to all hoki steak soups, imparting the characteristic fatty aroma of the fish. Its highest relative content in CZ 2 enhances the meaty flavor. n‐Nonanal and Decanal, typically associated with earthy notes in fish broths, exhibited the lowest levels in CZ, particularly in CZ 2, where both were significantly reduced compared to other hoki steak soups (*p* < 0.05). Furthermore, n‐Octanal and Heptanal, recognized as indicative compounds of fishy odor (Hu et al. 2022), were also notably diminished in CZ 2. Thus, the low fishy flavor of CZ 2 indicates that frying has some effect on the improvement of the flavor of hoki steak soups. Moreover, due to their high detection thresholds, alcohols and ketones exhibited ROAVs below 1, thereby exerting minimal influence on the overall flavor profile of hoki steak soups.

**TABLE 2:** Qualitative analysis of volatile compounds in hoki steak soups prepared by different processing methods.

Count	Compound	CAS#	Formula	RI	Rt (s)	Dt (ms)
1	n‐Nonanal (M)	C124196	C_9_H_18_O	1104.8	774.706	1.47463
2	n‐Nonanal (D)	C124196	C_9_H_18_O	1104.0	772.863	1.93812
3	2‐Nonanone	C821556	C_9_H_18_O	1094.2	750.75	1.40474
4	(E)‐2‐Octenal	C2548870	C_8_H_14_O	1066.6	691.782	1.33059
5	n‐Octanal (M)	C124130	C_8_H_16_O	1012.1	588.49	1.40187
6	n‐Octanal (D)	C124130	C_8_H_16_O	1011.6	587.529	1.81384
7	2‐Pentyl furan	C3777693	C_9_H_14_O	994.3	557.261	1.25219
8	3‐Octanone	C106683	C_8_H_16_O	990.7	549.574	1.30703
9	3‐Octanone (D)	C106683	C_8_H_16_O	990.4	549.093	1.71011
10	Benzaldehyde	C100527	C_7_H_6_O	978.6	525.071	1.15291
11	(E)‐2‐Heptenal (M)	C18829555	C_7_H_12_O	959.8	489.038	1.2596
12	(E)‐2‐Heptenal (D)	C18829555	C_7_H_12_O	958.8	487.117	1.66565
13	Heptanal (M)	C111717	C_7_H_14_O	898.9	388.279	1.33218
14	Heptanal (D)	C111717	C_7_H_14_O	900.1	389.928	1.69293
15	2‐Heptanone (M)	C110430	C_7_H_14_O	888.6	373.853	1.25446
16	2‐Heptanone (D)	C110430	C_7_H_14_O	886.4	370.967	1.62694
17	(E)‐2‐Hexenal (M)	C6728263	C_6_H_10_O	848.4	325.201	1.17868
18	(E)‐2‐Hexenal (D)	C6728263	C_6_H_10_O	849.1	325.922	1.51419
19	(Z)‐2‐Pentenol	C1576950	C_5_H_10_O	785.5	261.408	0.94506
20	3‐Methyl‐2‐butenal	C107868	C_5_H_8_O	785.5	261.408	1.09045
21	1‐hexanal (M)	C66251	C_6_H_12_O	788.6	264.291	1.26318
22	1‐hexanal (D)	C66251	C_6_H_12_O	788.6	264.291	1.5639
23	1‐Pentanol	C71410	C_5_H_12_O	769.6	245.549	1.25199
24	(E)‐2‐Pentenal (M)	C1576870	C_5_H_8_O	748.9	226.087	1.11406
25	(E)‐2‐Pentenal (D)	C1576870	C_5_H_8_O	747.7	225.006	1.36259
26	2‐Ethylfuran (M)	C3208160	C_6_H_8_O	696.6	183.558	1.04571
27	2‐Ethylfuran (D)	C3208160	C_6_H_8_O	699.5	185.721	1.3017
28	1‐Penten‐3‐one	C1629589	C_5_H_8_O	682.5	174.908	1.08299
29	Butanal	C123728	C_4_H_8_O	603.4	141.75	1.10163
30	Acetone	C67641	C_3_H_6_O	482.7	102.826	1.12649
31	Ethanol	C64175	C_2_H_6_O	476.0	101.023	1.05566
32	1‐Hexanol	C111273	C_6_H_14_O	879.1	361.702	1.32445
33	3‐Hexanone (M)	C589388	C_6_H_12_O	773.8	249.683	1.17203
34	3‐Hexanone (D)	C589388	C_6_H_12_O	771.8	247.698	1.47005
35	1‐Penten‐3‐ol (M)	C616251	C_5_H_10_O	694.7	182.195	0.94266
36	1‐Penten‐3‐ol (D)	C616251	C_5_H_10_O	686.4	176.736	1.34325
37	n‐pentanal (M)	C110623	C_5_H_10_O	696.1	183.187	1.19626
38	n‐pentanal (D)	C110623	C_5_H_10_O	696.4	183.435	1.4224
39	2‐Pentanone (M)	C107879	C_5_H_10_O	664.6	166.811	1.12196
40	2‐Pentanone (D)	C107879	C_5_H_10_O	661.8	165.57	1.36506
41	2‐Butanone	C78933	C_4_H_8_O	585.9	135.3	1.07269
42	2,4‐Dimethylbenzaldehyde	C15764166	C_9_H_10_O	1197.0	1018.427	1.2666
43	Decanal	C112312	C_10_H_20_O	1186.3	986.628	1.53683
44	2‐phenylacetaldehyde	C122781	C_8_H_8_O	1045.8	650.257	1.26231
45	6‐Methyl‐5‐hepten‐2‐one	C110930	C_8_H_14_O	991.7	551.731	1.18606
46	(Z)‐4‐Heptenal	C6728310	C_7_H_12_O	897.4	385.967	1.14609
47	2‐Methyl‐1‐butanol	C137326	C_5_H_12_O	738.9	217.277	1.23364

Abbreviations: Dt, drift time; MW, molecular mass; RI, retention index; Rt, retention time.

## 4 Conclusion

The study investigated the flavor compounds in hoki steak soups prepared through various processing methods employing GC‐IMS, E‐nose, E‐tongue, and combined with FAAs. Both E‐nose and GC‐IMS analyses revealed the dominance of aldehydes and alcohols in the hoki steak soups. GC‐IMS detected a total of 47 volatile compounds. n‐Pentanal (M) was identified as the key differential compound in the hoki steak soups based on VIP > 1. Additionally, six compounds, namely n‐Nonanal, n‐Octanal, Heptanal, 1‐Hexanal, Decanal, and (Z)‐4‐Heptenal, were screened with ROAV ≥ 1. E‐tongue combined with FAAs indicated that hoki steak soup's freshness improved after 2 h of cooking with oil. All four methods effectively differentiated the flavors of hoki steak soups prepared through different processing methods. Furthermore, the quality of hoki steak soups was assessed using pH, TBARS, and color, revealing that boiling for 2 h after stir‐frying enhanced the soup's quality. Collectively, these analyses demonstrated that the flavor and quality of the hoki steak soup improved significantly when boiled for 2 h after stir‐frying compared to other preparation methods.

## Author Contributions

**Xiaoqing Miao:** data curation (equal), methodology (equal), validation (equal). **Jing Li:** investigation (equal), software (equal). **Shuang Li:** formal analysis (equal), methodology (equal). **Guodong Li:** resources (equal). **Xiuping Dong:** supervision (equal). **Pengfei Jiang:** formal analysis (equal), funding acquisition (equal), project administration (equal), writing – original draft (equal), writing – review and editing (equal).

## Conflicts of Interest

The authors declare no conflicts of interest.

## Data Availability

All data generated or analyzed during this study are included in this manuscript.
